# Poly(A)-seq: A method for direct sequencing and analysis of the transcriptomic poly(A)-tails

**DOI:** 10.1371/journal.pone.0234696

**Published:** 2020-06-16

**Authors:** Fengyun Yu, Yu Zhang, Chao Cheng, Wenqing Wang, Zisong Zhou, Wenliang Rang, Han Yu, Yaxun Wei, Qijia Wu, Yi Zhang

**Affiliations:** 1 Laboratory for Genomics Regulation and Human Health, ABLife Inc., Wuhan, PR China; 2 ABLife BioBigData Instibute, Wuhan, PR China; 3 Center for Genomics Analysis, ABLife Inc., Wuhan, PR China; Wistar Institute, UNITED STATES

## Abstract

Poly(A) tails at the 3’ end of eukaryotic messenger RNAs control mRNA stability and translation efficiency. Facilitated by various NGS methods, alternative polyadenylation sites determining the 3ʹ-UTR length of gene transcripts have been extensively studied. However, poly(A) lengths demonstrating dynamic and developmental regulation remain largely unexplored. The recently developed NGS-based methods for genome-wide poly(A) profiling have promoted the study of genom-wide poly(A) dynamics. Here we present a straight forward NGS-method for poly(A) profiling, which applies a direct 3’-end adaptor ligation and the template switching for 5’-end adaptor ligation for cDNA library construction. Poly(A) lengths are directly calculated from base call data using a self-developed pipeline pA-finder. The libraries were directly sequenced from the 3ʹ-UTR regions into the followed poly(A) tails, firstly on NextSeq 500 to produce single-end 300-nt reads, demonstrating the method feasibility and that optimization of the fragmented RNA size for cDNA library construction could detecting longer poly (A) tails. We next applied Poly(A)-seq cDNA libraries containing 40-nt and 120-nt poly(A) tail spike-in RNAs on HiSeq X-ten and NovaSeq 6000 to obtain 150-nt and 250-nt pair-end reads. The sequencing profiles of the spike-in RNAs demonstrated both high accuracy and high quality score in reading poly(A) tails. The poly(A) signal bleeding into the 3’ adaptor sequence and a sharp decreased quality score at the junction were observed, allowing the modification of pA-finder to remove homopolymeric signal bleeding. We hope that wide applications of Poly(A)-seq help facilitate the study of the development- and disease-related poly(A) dynamics and regulation, and of the recent emerging mixed tailing regulation.

## Introduction

In eukaryotes, most messenger RNAs and some non-coding RNAs such as long non-coding RNAs and primary microRNAs undergo polyadenylation at the 3’ end of the transcripts, which serves as a mechanism for RNA stability and processing efficiency [1–4]. Recent applications of transcriptome-wide techniques have revealed the presence of multiple polyadenylation sites for most eukaryotic genes, and that the biosynthesis of poly(A) tails often concur with the selection of the poly(A) site in the nucleus [5–7]. Dynamic poly(A) tail regulation in the nucleus and cytoplasm has been implicated in various cellular and physiological processes, including embryonic development [8], cellular senescence [9, 10], inflammation [11] and various diseases [4]. Widespread uridylation and guanylation at the downstream of poly(A) tails has been recently revealed. Uridylation is reported to decrease mRNA stability, while the mixed tailing tends to increase mRNA stability [12–14].

Despite the importance of mRNA polyadenylation, until a couple years ago we knew surprisingly little about the global features of poly(A) tails. Almost two decades ago methods based on Northern Blot and/or 3’ RACE had been developed to assay the length of poly(A) tails [15, 16], followed by the Sanger sequencing-based method [17], all of which were used for the investigation of individual genes. With the arrival of the era of next-generation sequencing (NGS), our knowledge of poly(A) tails rapidly falls behind the knowledge of RNA sequences in general. High-throughput sequencing techniques are believed unfavorable to read through long homopolymeric sequences. Traditional library generation and mapping strategy also bias against poly(A) tails [18].

Two NGS-based techniques that came out in 2014 proposed solutions to this problem. Chang and colleagues introduced TAIL-seq [14]. The cDNA libraries made from the poly(A) mRNA were sequenced from the 3’ termini to mRNA bodies. To overcome the low quality of the sequencing reads from the 3’-end of the poly(A) tails, Chang et al. developed a special algorithm to determine the exact boundary between the poly(A) tail and the mRNA body, allowing the measurement of global poly(A) lengths. In another paper, Subtelny et al. approached the subject with PAL-seq technique [8]. After generating a similar poly(A) mRNA enriched library, instead of directly sequencing the poly(A) region, they used primer extension reaction to incorporate dTTP and biotin-dUTP, and then detected the signals by incubating with fluorescent streptavidin. Poly(A) tail lengths were calculated with normalized fluorescent intensities. While it was believed that mammalian mRNA typically contains poly(A) tails over 200 nt [19, 20], the median poly(A) length for human HeLa cells was reported to be 67 nt by PAL-seq [8] and 60 nt by TAIL-seq [14], and for mouse NIH 3T3 cells the median poly(A) length is 96 nt and 61 nt measured by the two methods, respectively [8, 14].

PAL-seq method requires modification of the sequencing platform, while TAIL-seq requires complicated data processing algorithm. Another NGS based method, PAT-seq, which was developed to measure poly(A) tail lengths and polyadenylation sites in the budding yeast *S*. *cerevisiae* [21], uses standard sequencing procedures to obtain poly(A) containing reads. The reported average poly(A) length in yeast is 25.6 nt [21], similar to results from PAL-seq data. However, PAT-seq has not been applied to measure mammalian poly(A) tails [21]. An enhanced version of TAIL-seq, mTAIL-seq has been reported, which efficiently reduces the required amount of input mRNAs [22]. Differently, TED-seq was later developed to estimate the poly(A) tail length of a transcript by subtracting the sequence 3ʹ-UTR length from the selected cDNA library size [23]. More recently, Nanopore sequencing [24], FLAM-seq [25], and PAlso-seq [26] have been developed to directly sequence the poly(A) tail length using single molecule sequencing technologies on Nanopore and PacBio platforms.

In this study, we aimed to develop a direct poly(A) sequencing method Poly(A)-seq. We developed a new poly(A)-enriched cDNA library construction protocol, as well as an algorithm pA-finder to identify poly(A)-containing sequence and calculate the median poly(A) tail length in genes. With the initial success on Illumina NextSeq 500, we next ran Poly(A)-seq cDNA library on the more popular HiSeq X Ten and NovaSeq 6000, with a focus on studying the poly(A) reading accuracy and the poly(A) detection power on two different sizes of poly(A) tail spike-in. We hope that the introduction of this more straightforward poly(A) profiling method can lead to more in-depth discoveries of poly(A) features.

## Materials and methods

### Cell culture

HeLa, HCT116 and HEK293T cells were incubated in Dulbecco’s modified Eagle’s medium (DMEM) with 10% newborn bovine serum plus 100 U penicillin/streptomycin (Hyclone) at 37°C in 5% CO_2_. The PTBP1 siRNA HeLa cell line was described in [27].

### Poly(A)-seq library generation and sequencing

Total RNA was extracted with Trizol and treated with RQ1 DNase (Promega) to remove DNA. The quality and quantity of the purified RNA were determined by measuring the absorbance at 260nm/280nm (A260/A280) using Smartspec Plus (BioRad). RNA integrity was further verified by 1.5% Agarose gel electrophoresis. Total RNA was then fragmented with RNase T1 at 37°C for 3 minutes. For each sample, 5.1 μg of fragmented total RNA was used for Poly(A)-seq library preparation. Polyadenylated mRNAs were captured with oligo(dT)-conjugated magnetic beads (Invitrogen). Furthermore, 3’ adaptors were ligated to the 3’ end of captured mRNAs using GnomeGen sRNA-seq library preparation kit. And then reverse transcription was performed with RT primer that was complemented with 3' adaptor, followed by synthesizing DNA with Terminal-Tagging oligo using ScriptSeq™ v2 RNA-Seq Library Preparation Kit (epicentre). The cDNAs were purified and PCR amplified by 18 cycles. PCR products corresponding to two size ranges, 250–350 bps and 300–500 bps, were purified, quantified and stored at -80°C until used. For high-throughput sequencing, the libraries were prepared following the manufacturer's instructions and applied to Illumina NextSeq 500 system for 300 nt single-end sequencing. The original sequencing data can be accessed at GSE84287.

### pA-finder: Poly(A)-seq data processing

The Poly(A)-seq data processing pipeline is outlined in Fig 2A. The base calls were acquired from NextSeq 500 using NextSeq Control Software 1.4. After trimming off poly(G) and 5’, 3’ adaptor sequences from Poly(A)-seq raw reads, we first searched for the poly(A) regions in the reads. We began the search by scanning the full reads and finding the first 9A sequence (allowing 0.1 error rate) closest to the 5’ end and 6A (allowing 0.2 error rate) closest to the 3’ end. The sequence in between was extracted as the preliminary poly(A) region, and reads without preliminary poly(A) region or with preliminary poly(A) < 10 nt were discarded. After extracting the preliminary poly(A) region, the remaining sequence towards the 5’ end of the reads was used for mapping to reference human genome (GRCH38). In the mapping step, at least 20 nt was required and Tophat2 was used allowing up to 2 mismatches. Reads that were uniquely mapped to human genome were kept for analysis of poly(A) length. Next we scanned the preliminary poly(A) region to label any consecutive non-A sequences > = 5 nt in length. The longest sequence within the region not containing consecutive non-As was determined as the poly(A) region and the length was counted. To determine whether a poly(A)-containing tag was fully sequenced, we scanned for the 3’ adaptor sequence downstream of the poly(A) region in the raw reads.

To eliminate the noise of the bleeding of homopolymer signal into the 3’ adaptor regions as mentioned previously [14], we added an additional filter. We identified the poly(A) end position showing a sharp decrease in the quality score where three consecutive As showing an average base quality score less than 25.

To compare poly(A) profiling results between Poly(A)-seq and published methods, we downloaded data for PAL-seq [8], TAIL-seq [14] and PAT-seq from the GEO database and applied our Poly(A)-seq data analysis pipeline to these data with minor modifications to accommodate for the difference in data properties. Reads from mice and *S*. *cerevisiae* samples were mapped to corresponding reference genomes GRCm38.p3 and S228C. Since PAL-seq data do not contain poly(A) information for each tag, we were unable to analyze the ratio of poly(A)-containing reads in these samples.

### Spike-in RNA preparation

DNA oligos containing a 40-nt poly(A) sequence (Tianyi Huiyuan Inc., Wuhan) and a 120-nt poly(A) sequence (IDT, Singapore) were chemically synthesized to generate poly(A)-tail-length standards.

The 40-nt poly(A) sequence is located between the 5’ end adaptor sequence (5’- TAATACGACTCACTATAGGGTTTAACGCGAATTAATTCTGTGGAATGTGTGTCAGTTAGG- 3’) and the 3’ end adaptor sequence (5’-CATTGCCTAGAGTCGGACTGA-3’). The 5’ end adaptor sequence contained the T7 promoter sequence and a segment of PcDNA3.1 plasmid sequence, and the 3’ end adaptor sequence contained sequence of BsrD1 cutting sites. The 120-nt poly(A) sequence is located between the 5’ end adaptor sequence (5’- TAATACGACTCACTATAGGGTCGACGCTCAAGTCAGAGGTGGCGAAACCCGACAGGACTA—3’) and the 3’ end adaptor sequence (5’-CATTGCCTAGAGTCGGACTGA-3’). The 5’ end adaptor sequence contained a 20-nt T7 promoter sequence which was followed by a 40-nt or 39-nt PcDNA3.1 plasmid sequence, and the 3’ end adaptor sequence contained a BsrD1 cutting site allowing the complete removal of the 3’ adaptor sequence prior to *in vitro* transcription.

The DNA templates for *in vitro* synthesis of the spike-in poly(A) tail RNA were amplified using the forward primer containing the T7 promoter sequence (5’- TAATACGACTCACTATAGGG-3’) and the reverse primer targeting 3’ end adaptor sequence (5’- TCAGTCCGACTCTAGGCA -3’). The PCR products were purified by VAHTS DNA Clean Beads (Vazyme, N411-03) and then cut with BsrD1 (NEB, R0574S) to remove the 3’ end adaptor sequences, leaving the 3’ poly(A) tail sequence at the very end of the template. The enzyme-digested product was gel purified using a Qiagen column kit after electrophoresis on a 4.0% agarose gel. The *in vitro* transcription was carried out using TranscriptAid T7 High Yield Transcription Kit (Thermo, K0441). The resulted 80-nt spike-in RNA (40-nt adaptor followed by 40-nt poly(A) tail) and 160-nt spike-in RNA (40-nt adaptor followed by 120-nt poly(A) tail) were purified on a 12% denaturing urea polyacrylamide gel.

### Poly(A)-seq on HiSeq X Ten and NovaSeq 6000 with total RNA from HeLa cells and Spike-in RNAs

For each sample, 5 μg of total RNA prepared from HeLa cells was used. Total RNA was fragmented by RNase T1 (Thermo, EN0541), then the poly(A)-containing RNA fragments were captured with oligo(dT)-conjugated magnetic beads (Invitrogen, 61005). The purified spike-in RNAs were then added to continue library preparation, which included 3' adaptor ligation (gnomegen, K02420-L) and reverse transcription with RT primer that was complemented with 3' adaptor. The 2^nd^ strand DNA was then synthesized using SMARTer® Stranded RNA-Seq Kit (TAKARA, 634837). The libraries were sequenced using the 150-nt paired-end kit on Illumina HiSeq X Ten or using the 250-nt paired-end kit on Illumina NovaSeq 6000 (Novogene, Beijing). The original sequencing data can be accessed at GSE84287.

During data analysis, the spike-in poly(A) reads were retrieved by aligning with the unique 5’ adaptor sequence using Tophat2. The length of poly(A) tail was calculated using pA-finder. For the 160-nt spike-in RNA containing 120-nt poly(A) tail sequence, its 150-nt sequencing reads from end 1 were composed of 6-nt of the library sequence, 39-nt of the 5’ adaptor sequence (unique to each spike-in) and 104-nt poly(A) sequence.

For analysis of the NovaSeq Poly(A)-seq reads, we ran pA-finder one time using default parameters, and another time using the additional filter by identifying the poly(A) end position showing a sharp decrease as described above.

### Poly(A) tail length measurement using PCR-based method

To validate the alteration of poly(A) length of individual genes, we used Poly(A) Tail-Length Assay Kit (Affymetrix, 76455) that is based on high resolution poly(A) tail (Hire-PAT) assay [14] as described in the manufacture’s instruction to measure poly(A) length in HeLa cell and HCT116 cell. In brief, total RNA was isolated and guanosine and inosine (G/I) residues were tailed to the 3’ end. The tailed-RNAs are reverse-transcribed using the newly added G/I tails as the priming sites. PCR amplification was performed using two primer sets: (1) a gene-specific forward and reverse primer set designed upstream of the polyadenylation site as a control and (2) a gene-specific forward primer and the universal reverse primer which is provided with the kit to generate a product including poly(A) length. Used gene-specific primers are as follows: NPM1 (forward, 5’-GTTGTCCAAAATGCCTGT-3’; reverse, 5’- ATACTGAGTTTTATTTCACATG-3’), ACTB (forward, 5’- AGAATGGCCCAGTCCTCTC-3’; reverse, 5’-AACTGGTCTCAAGTCAGTGTAC-3’), and GAPDH (forward, 5’-GCAAGAGCACAAGAGGAAGAG-3’; reverse, 5’- AACTGGTTGAGCACAGGGTA-3’). PCR products were detected by polyacrylamide gel electrophoresis.

The poly(A) lengths of the *in vitro* transcribed 40-nt poly(A)-tail spike-in RNAs were also confirmed by the same method using the spike-in backbone sequence-specific forward primer 5’- GTGGAATGTGTGTCAGTTAGG-3’.

## Results and discussion

### Poly(A)-seq library generation and NextSeq 500 sequencing strategy

**Fig 1:**
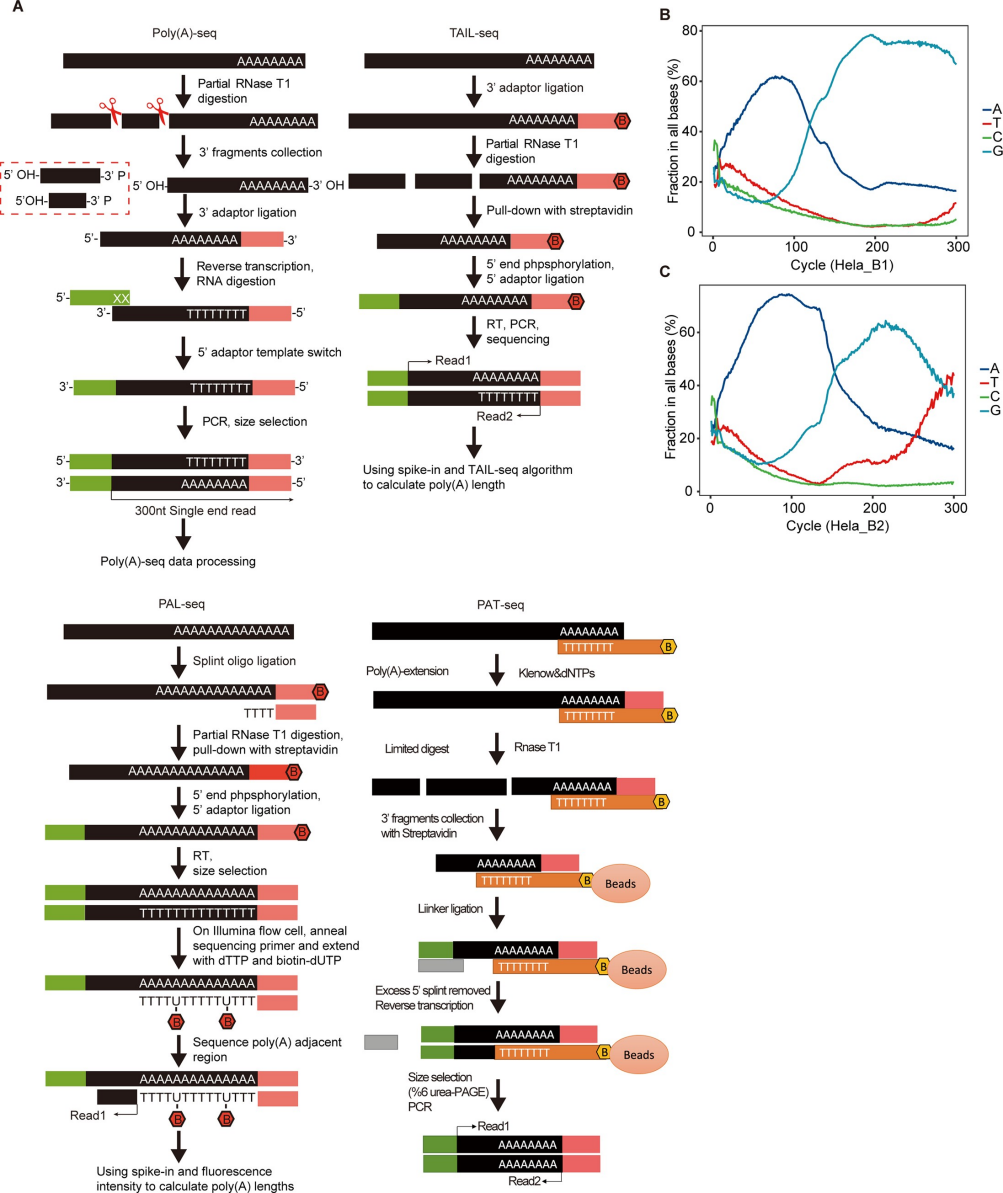
Poly(A)-seq library construction and the NextSeq 500 sequencing profiles. A. Flowchart of Poly(A)-seq, TAIL-seq, PAL-seq, and PAT-seq library construction procedures. B. Distribution of four different sequenced bases in each of the 300 cycle of sequencing on Illumina NextSeq 500 for sample Hela_B1. C. Distribution of four different sequenced bases in each of the 300 cycle of sequencing on Illumina NextSeq 500 for sample Hela_B2.

Currently, there are various methods for genome-wide mapping of the polyadenylation sites, such as PAS-seq [5], PAT-seq [21] and 3’P-seq [28]. PAT-seq data was also used to deduce the short poly(A) tail lengths in yeast cells [21]. TAIL-seq and PAL-seq published for specific poly(A) length measurement both obtained 51 nt (TAIL-seq) [14] or 36 nt (PAL-seq) [8] 5’-end reads for mapping each cDNA onto specific genes (Fig 1A, top-right and bottom-left panels). These two methods are divergent in quantifying the poly(A) length in each cDNA tag: PAL-seq applied hybridization approach and TAIL-seq sequenced the poly(A) end from the 3’ adaptor (Fig 1A).

To more robustly measure poly(A) lengths at genome-wide level in mammalian cell transcriptomes, we decided to build a straight forward Poly(A)-seq method for one-round sequencing of the cDNA tags containing the complementary sequence of both poly(A) tail and its upstream genomic sequence of a specific mRNA/lncRNA gene (Fig 1A, top-left).

In Poly(A)-seq, we applied a partial RNase T1 digestion of total RNAs to yield a 3’-phosphate group at all RNase cleaved sites, which was similar to both PAL-seq and TAIL-seq. This treatment ensured that the followed 3’-end adaptor ligation occurs at the natural 3’-OH ends of all transcripts, but not at the digested 3’-phosphate ends. Secondly, we added the 3’-end adaptor to the digested smaller RNA fragments, which should be more efficient than directly adding to the non-digested RNAs, according to the previous report [29] (Fig 1A top-left). This strategy differs from those of PAL-seq and TAIL-seq. Given that mammalian poly(A) tails are generally about 200-nt in length [11, 20], we selected RNase T1 concentration and digestion condition yielding RNA fragments around 200–300 nt for cDNA library construction, based on the digestion profile on agarose gel. The poly(A)-enriched and 3’ adaptor-ligated RNA fragments were then used to template the synthesis of the first strand cDNA. We would like to point out that we have applied oligo(dT) beads to enrich poly(A) sequence-containing RNAs after RNase T1 digestion, which could be more efficient in enriching the long-tailed RNAs than the short-tailed RNAs. This bias may influence the detected tail length.

The second strand was synthesized using the 5’-end adaptor-containing primer and a template switch protocol, which has been proven of high efficiency [30]. The cDNA library was PCR amplified and sequenced on Illumina platforms from the 5’-end adaptor using the first cDNA strand as template, which was completed with the regular base calling step (Fig 1A, top-left).

In summary, the library construction protocol of Poly(A)-seq is different from the recently published PAT-seq protocol (Fig 1A, bottom-right) which adapted the ePAT protocol [31] in the following aspects. The library construction protocol is different in the capture of the poly(A) tail-containing RNA fragments, RNase T1 partial digestion, and 5’-end priming and synthesis of the second strand of cDNA. The major differences between Poly(A)-seq and TAIL-seq strategy also lie in the library construction and sequencing strategy (Fig 1A). In Poly(A)-seq, the reading is from the 5’ adaptor and all the way down to the 3’ end of the cDNAs, and from the 5’-mRNA body towards the poly(T) sequence on the cDNA templates, which finally reaches the 3’ adaptor sequence (Fig 1A).

In this study, we performed four Poly(A)-seq runs on the Illumina NextSeq 500 platform and achieved consistently acceptable percentages of high-quality reads. The pass-filter cDNA clusters were above 63% in all four runs (Table 1), and the percentages of high-quality reads (Q> = 30) were consistently over 46% (Table 1). After the initial reading of several cycles, the A-ratio (darkblue line) started to increase while those of the other three bases decreased, consistent with the prevalence of poly(T) sequence in the cDNA tags (Fig 1B and 1C). In one sequencing run, the A-ratio reached up to over 60% at about 60 cycles and gradually declined around 100 cycles (Fig 1B).

**Table 1:** Poly(A)-seq sequencing quality scores from NextSeq 500.

Sequencing run	Mix conc. (ng/μL)	Mix conc. (nM)	DNA conc. (pM)	Density (K/mm2)	Cluster Pass Filter (%)	%> = Q30
1	1.13	4.68	2.2	238	71.62	56.40
2	1.48	7.4	2.2	274	66.73	46.90
3	2.4	10.68	1.80	205.75	63.50	46.50
4	2.14	9.53	2.00	205.25	77.03	46.00

For Illumina NextSeq 500 sequencing platform, the increase of G-ratio (blue line) indicates the loss of reading signals. We found that at the time when A-ratio was peaked after 70-cycle, G-ratio starts to increase which reached over 78% at cycles 192–197. After cycle 160, the G-ratio and A-ratio were gradually stabilized at around 70% and 20%, respectively (Fig 1B, upper). The similar base dynamics were observed in another sequencing bench (Fig 1B, lower). We speculated that the reading signal loss could be due to the fall off of the sequencing polymerase either when it reached the end of short cDNAs. Alternatively, the sequencing polymerase could fall off the cDNA template during synthesis of long homopolymeric poly(A) tails from the poly(T) template. The sustained 20% A signal could be resulted from the portion of high-processive DNA polymerase.

### pA-finder: Extracting poly(A) tails from Poly(A)-seq and PAT-seq data

**Fig 2:**
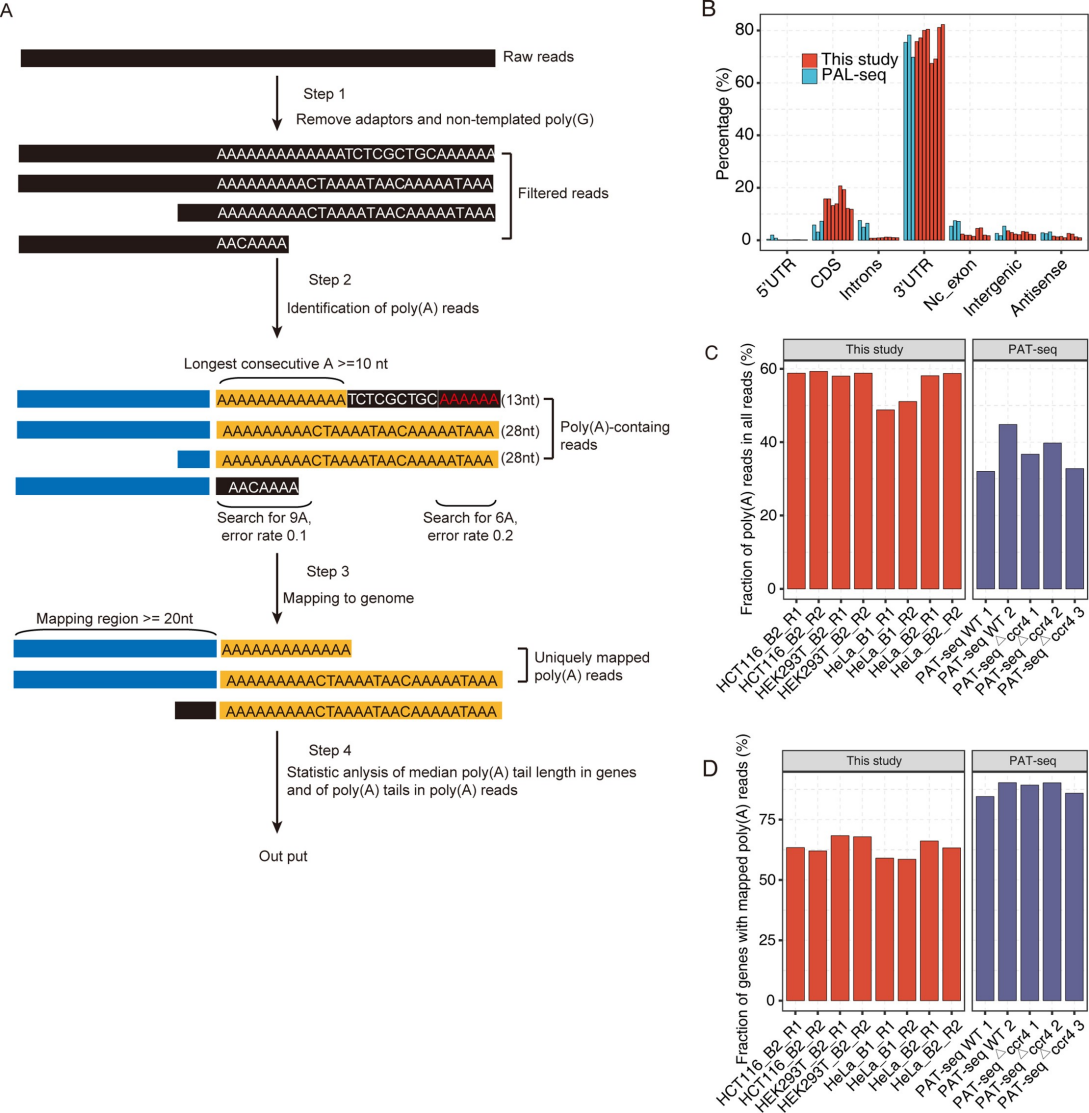
pA-finder identification of the poly(A)-containing reads and the poly(A) tail length of each gene. A. Flowchart of pA-finder. B. Genome distribution of the uniquely mapped reads in Poly(A)-seq, PAL-seq and TAIL-seq samples. C. Percentage of poly(A)-containing reads out of all raw reads of Poly(A)-seq and PAT-seq samples. D. The number of genes with poly(A)-containing reads detected by Poly(A)-seq and PAT-seq.

After sequencing, we used a self-developed algorithm called pA-finder to analyze the raw reads and determine the poly(A) length for each sequenced cDNA tag and the median poly(A) length for a specific gene. Step 1, the adaptor and non-templated poly(G) sequences were removed from the raw sequencing reads (Fig 2A). Step 2, we located the 5’-start and 3’-end of a poly(A) tail in each cDNA tag by searching for the appearance of a 9-A and 6-A segments from the 5’-end and 3’-end, respectively (Fig 2A). Given the relative high sequencing error in reading the poly(A) sequence, we allowed 1 mismatch for each search. Step 3, once the putative poly(A) tail of a cDNA tag was determined, its upstream anchor sequence was mapped to specify each cDNA tag to the gene that it was derived. Step 4, The quality of the poly(A) tail was further assured by two additional criteria, which led to the identification of the poly(A) tail length in each poly(A)-containing read. First, the poly(A) tail must contain 10 consecutive poly(A) sequence without mismatch. Second, if a poly(A) tail contained a continuous non-A sequence longer than 4-nt, only the longest consecutive poly(A) segment was regarded as the poly(A) tail sequence. In this way, the internal poly(A) contamination originating from the genome-encoded continuous As could be largely removed if there was any. Step 5, Statistic analysis of the median poly(A) length of each gene based on lengths of all the poly(A) tails detected inside of a specific gene.

To compare the data obtained using Poly(A)-seq strategy with those using other strategies, we downloaded and re-analyzed datasets for mammalian cells from PAL-seq [8] and TAIL-seq [14] methods, and also a set of PAT-seq for yeast cells [21] that was used for analyzing polyadenylation sites rather than poly(A) length. Nevertheless, the first cDNA strand (read 1) datasets from PAL-seq [8] and TAIL-seq [14] methods were only usable in analyzing for their genomic distribution, but not for the poly(A) length dynamics. We included PAT-seq read 1 to test the robustness of the pA-finder pipeline.

PAL-seq first cDNA strand datasets contained 8.10–20.49 millions of high-quality reads for each sample, and TAIL-seq provided two sequencing data 5.26–8.37 millions of high-quality reads from the first cDNA strand (S2 Table). Five PAT-seq data contained 5.59–10.7 millions of yeast transcriptomic reads. In this study, a total of 8 Poly(A)-seq libraries were generated and sequenced from the first cDNA strand for 300 cycles. These libraries were from HeLa, HCT116 and HEK293T cells, respectively, and each of them obtained 9.34–20.90 millions of high-quality reads (S1 Table). Mapping of the high-quality reads from the first cDNA strand after removing non-template poly(A) tails revealed that Poly(A)-seq, PAL-seq and PAT-seq reads were highly enriched in 3ʹ-UTR as expected, while TAIL-seq reads were not (Fig 2B, S2 Table).

We next ran pA-finder to extracted poly(A) sequence information from the first cDNA strand of Poly(A)-seq and PAT-seq reads. We demonstrated that about 50–60% of Poly(A)-seq reads from mammalian cells harbored detectable poly(A) tails (> = 11nt) in ~15000 genes (Fig 2C, S3 Table), and about 30–45% PAT-seq reads harbored detectable poly(A) tails in ~5000 genes (Fig 2C and 2D, S3 Table). These results confirmed that Poly(A)-seq strategy of reading poly(A) sequence from the 5’ adaptor and first-strand of cDNA was successful.

### Poly(A)-seq detected poly(A) tails in mammalian cells are peaked around 100 nt

**Fig 3:**
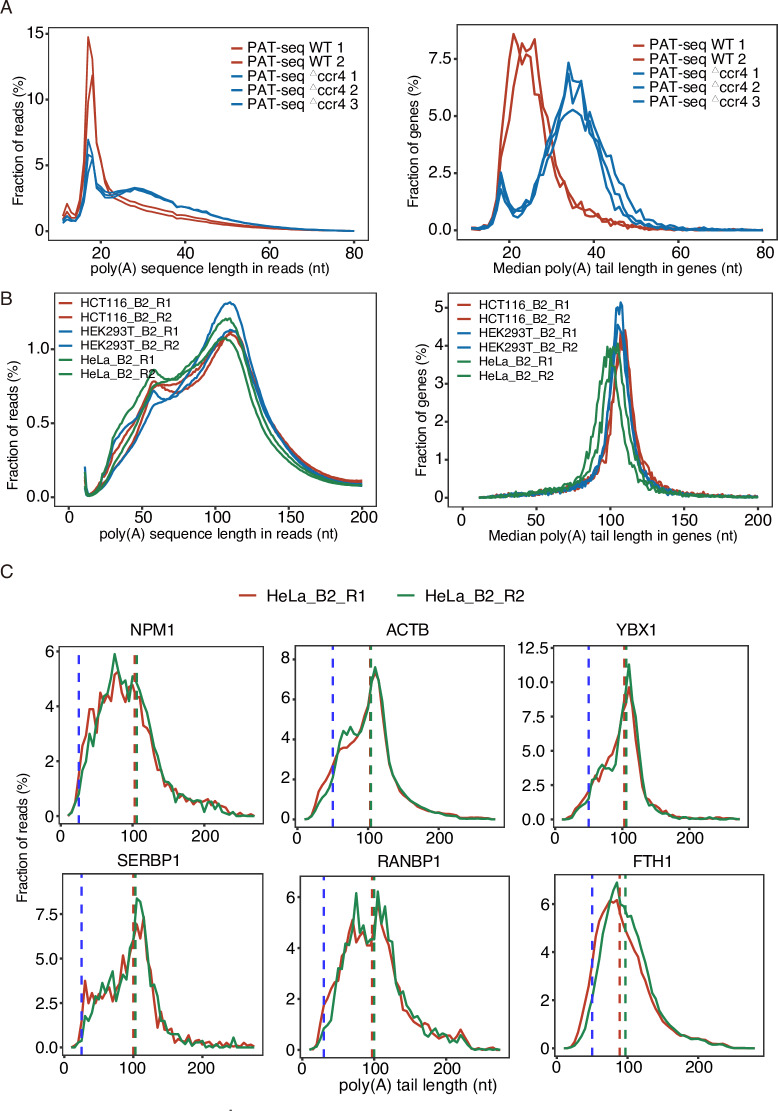
The length profiles of poly(A) sequence in Poly(A)-seq reads and of median poly(A) tails in genes. A. The poly(A) tail length distribution in reads (left) and genes (right) in yeast, which is resulted from the analysis of PAT-seq data [21] by running pA-finder. B. The poly(A) tail length distribution in reads (left) and genes (right) in three human cell lines, which is resulted from the analysis of Poly(A)-seq data in this study by running pA-finder. C. Poly(A) tail profiles in poly(A)-containing reads of 6 human genes in HeLa cells. The blue dashed line indicates the poly(A) tail length detected by TAIL-seq [14]. The red and green dashed lines indicate the median poly(A) tail length of HeLa_B2_R1 and HeLa_B2_R2, respectively.

Statistic analysis of the poly(A) tail length in poly(A) reads showed that PAT-seq detected poly(A) sequence were peaked at 16–20 nt in length in poly(A) reads (Fig 3A, left). The mean poly(A) tails calculated based on the poly(A) reads revealed that the poly(A) tails of wild-type yeast were peaked at around 18–33 nt, similar to those detected by PAL-seq [8]. In the ccr4 mutant yeast cells, the poly(A) tails in yeast were shifted to 27–44 nt (Fig 3A, right). These results were consistent with those reported by Harrison *et al* [21].

We then analyzed the poly(A) tails detected by Poly(A)-seq performed on three different mammalian cell lines, showing a broad peak ranging from 81–118 nt in length (Fig 3B). However, the population of poly(A) tails longer than 150-nt was difficult to be detected by Poly(A)-seq. When compared the Poly(A)-seq results with previously reported human cell poly(A) profiles by PAL-seq and TAIL-seq, we found that poly(A) tails detected by Poly(A)-seq were longer. For example, the median poly(A) tail lengths were 93–98 nt for HeLa cells, 101–102 nt for HCT116 cells, and 100–103 nt for HEK293T cells (Table 2). These median poly(A) tail lengths were much longer than the 59 nt (HeLa) detected by TAIL-seq [14] and 67.5 nt (HeLa) and 75.3 nt (HEK293T) detected by PAL-seq techniques [8]. The potential reasons for the longer poly(A) tails detected by Poly(A)-seq method were further described below.

**Table 2:** Poly(A)-seq profiles of human cells.

Sample	Total reads	Total genes	Median poly(A) length	Sequencing platform	Run
Bench 1					
HeLa_B1_*R*1	11292091	13490	60	Nextseq 500	1,2
HeLa_B1_*R*2	11805400	13306	63		1,2
Bench 2					
HeLa_B2_*R*1	12846233	15656	93	Nextseq 500	3,4
HeLa_B2_*R*2	8879440	14482	98		3,4
HCT116_B2_*R*1	10695232	14761	101		3,4
HCT116_B2_*R*2	12543975	14403	102		3,4
HEK293T_B2_*R*1	14640822	16487	100		3,4
HEK293T_B2_*R*2	13729515	16240	103		3,4
Spike-in					
HeLa_1.A40	20935	spike-in	44	HiSeq X Ten	5
HeLa_2.A40	6478	spike-in	44		5
HeLa_1.A120	85531	spike-in	105*		5
HeLa_2.A120	23827	spike-in	105*		5
HeLa_1.A40	21821	spike-in	42	NovaSeq 6000	6
HeLa_2.A40	7815	spike-in	42		6
HeLa_1.A120	59179	spike-in	112		6
HeLa_2.A120	18404	spike-in	109		6
Cells					
HeLa_1	5303467	11352	67	HiSeq X Ten	5
HeLa_2	6246736	11300	65		5
HeLa_1	8419838	11950	54	NovaSeq 6000	6
HeLa_2	10385586	12015	53		6

Total reads: Total number of uniquely mapped reads that contain poly(A) tails > = 10 nt.

Total genes: Total number of genes with > = 1 poly(A)-containing reads.

% 3’ adaptor reads: Percentage of total reads that contain 3’ adaptor sequences downstream of poly(A) regions.

Median poly(A) length: Median poly(A) length in total reads.

“B*” in sample name means different bench. “R*” means replicates.

We next compared the poly(A) length profiles in poly(A) reads of some specific genes detected both by Poly(A)-seq and TAIL-seq (Fig 3C). In general, the poly(A) tails detected by Poly(A)-seq were longer than those detected by TAIL-seq (S2A Fig).

To explore whether the longer poly(A) tails detected by Poly(A)-seq in this study than what were reported by TAIL-seq and PAL-seq were due to the pA-finder mistakenly including any 3’-UTR regions in the poly(A) sequences, we mapped the position of the last nucleotide immediately before the 5’ boundary of the pA-finder detected poly(A), demonstrating that that the majority of the detected polyadenylation sites were mapped to the annotated polyadenylation sites (TSS, transcription termination site), which did not vary among different tail-length bins (S1 Fig).

### Increase of the poly(A) tail detection power by increasing the sizes of RNA fragments and cDNA libraries

Poly(A) tails in mammalian cells are generally considered around 200 nt in length when during mRNA maturation in nucleus. The tail may be shortened after exported into cytoplasm due to the deadenylation. An important step in preparing DNA or RNA samples for NGS analysis is to fragment sequences to a target length for cDNA library construction, and a favorable library size was chosen for sequencing. In this study, we speculated that it could be possible to detect longer poly(A) tails by optimizing the sizes of RNA fragments and cDNA libraries.

**Fig 4:**
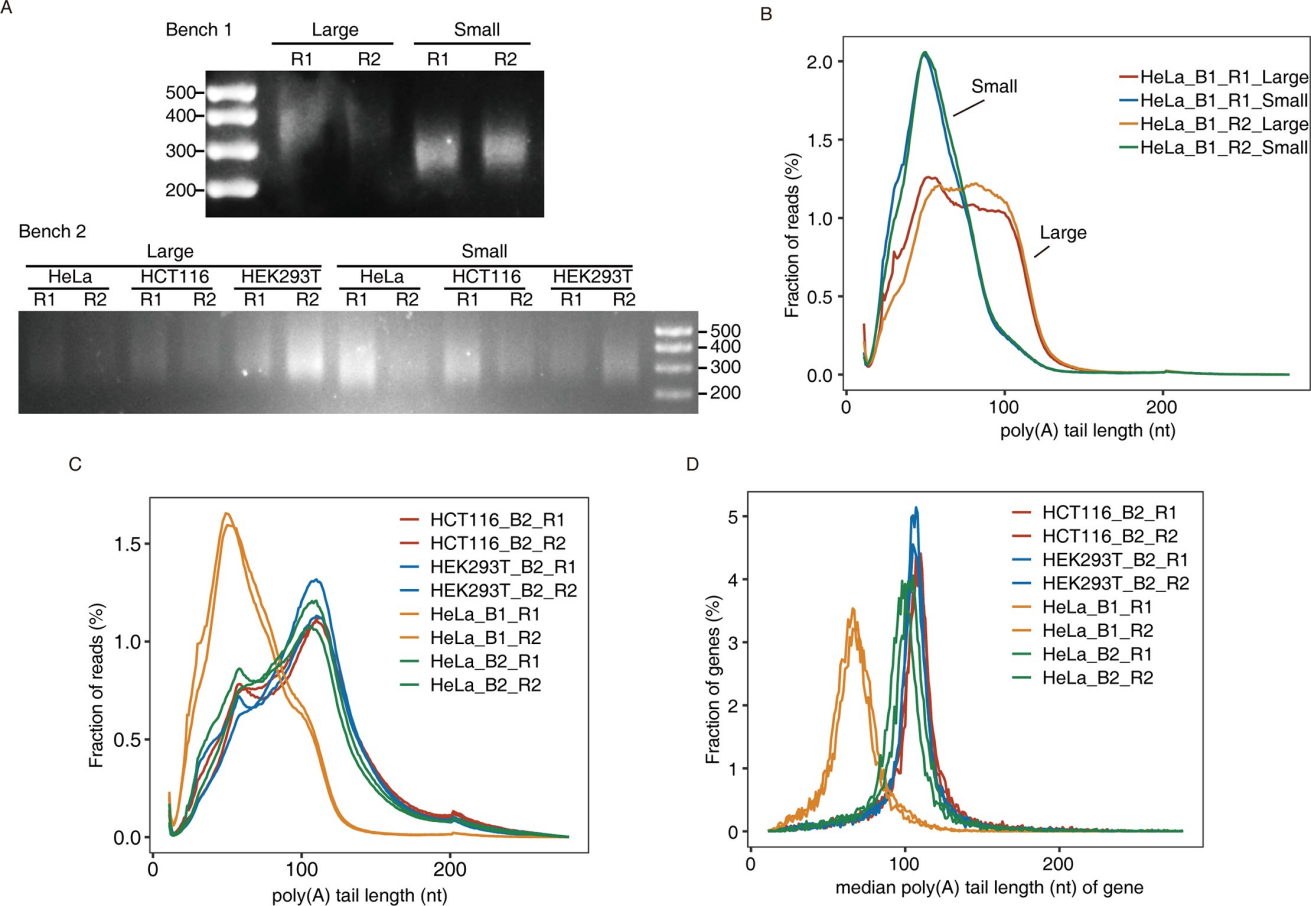
Increase of the detected poly(A) tail lengths by sequencing the larger size of cDNA library and by reducing the size of RNA fragments. A. Electrophoresis gel images of selected cDNA libraries for Poly(A)-seq in two benches of experiments. In experiment 1, after size selection, we separated two different selection products on an agarose gel. “Large” and “Small” were significantly different in sizes, indicating the inserted RNA fragments were not homogenized. In experiment 2, no much difference between these two selections was observed, indicating the inserted RNA fragments were homogenized and smaller. B. Distribution of poly(A) tail length in poly(A)-containing reads in Large and Small cDNA libraries in the experiment 1. C. Poly(A) tail length distribution in reads across all samples. D. Distribution of median poly(A) tail lengths in all genes across all samples.

To test whether cDNA libraries affected the detected poly(A) length, we kept two different sizes 350–450 nt (Large) and 250–350 nt (Small) from the same set of Poly(A)-seq cDNA libraries generated with HeLa cells during the size selection step for Illumina sequencing (Fig 4A, upper). Both the large and small-size cDNA libraries produced roughly the same number of uniquely mapped reads (Table 3, S1 Table), however, poly(A) lengths detected in poly(A) reads in the large-size cDNA libraries were obviously longer (~30–120 nt) than those detected in the small-size libraries (~30–80 nt) (Fig 4B, Table 3), indicating that cDNA libraries with longer inserts are more enriched in longer poly(A) sequences.

**Table 3:** Poly(A)-seq profiles of Large and Small libraries.

Sample	Total reads	Total genes	Median poly(A) length	Run
HeLa_B1_R1 Large	5506526	14067	71	1
HeLa_B1_R1 Small	5785565	13865	53	2
HeLa_B1_R2 Large	5785797	14133	75	1
HeLa_B1_R2 Small	6019603	13640	55	2

Total reads, total genes, %Median poly(A) length: same as Table 2.

Next, the reads from Large and Small libraries were combined for further analysis of poly(A) tail profiles in poly(A) reads (Fig 4C) and of the median poly(A) tail lengths for genes (Fig 4D). Interestingly, the median length of poly(A) tails was 60 nt in the first bench of HeLa 1 (Table 2), comparable with results by TAIL-seq (59 nt) [14] and PAL-seq (67.5 nt) techniques [8].

Given that Poly(A)-seq sequences poly(A)-containing reads from the upstream toward poly(A) tail, we speculated that when the library sizes were constrained by sequencing platform, the longer the RNaseT1 RNA fragments were inserted, the shorter the poly(A) sequence could be included in a poly(A) cDNA molecule. To increase the detected poly(A) tail length, we thusly reduced the size of RNA fragments by increasing RNase T1 concentration. We extracted total RNAs from HeLa cells, HCT116 and HEK293T cells. Two experimental repeats for each cell line were included for constructing Poly(A)-seq cDNA libraries and sequencing (Table 3, S1 Table). The poly(A) sequence lengths in poly(A) reads were highly consistent between experimental repeats and among cell lines, which were significantly increased when compared to the first bench of experiments (Fig 4C and Table 3). We then calculated the median poly(A) length for each gene. The detected poly(A) tails in three different cell lines by the second experiments were close to each other, peaking around 100 nt, which were much longer than the first bench of experiments as well (Fig 4D, Table 3). This success suggested that modification of the length of RNA inserts represents an effective way to expand the detected poly(A) tail length, and accurate profiling of the complete poly(A) tails could be possible.

To access the reliability of the poly(A) tails obtained in this study among different sets of experiments and those obtained by TAIL-seq and PAL-seq, particularly in HeLa cells, we found very poor correlation among the tail sizes obtained by different methods, and a better correlation among samples within Poly(A)-seq method (S2 Fig).

### Validation of the Poly(A)-seq detected poly(A) tails

**Fig 5:**
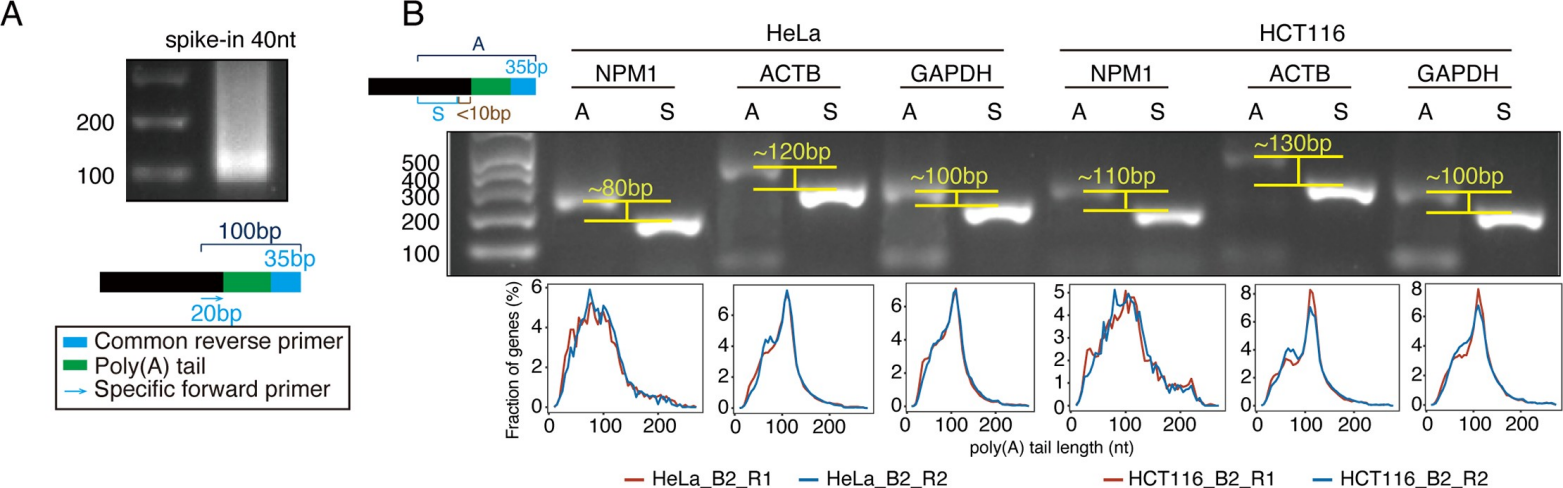
Hire-PAT PCR assay of poly(A) tail length obtained by Poly(A)-seq method. A. Poly(A) tail length validation of the 40-nt spike-in using Hire-PAT PCR assay. PCR product was showed in top panel and the experiment was characterized in the bottom panel. B. Poly(A) tail length of NPM1, ACTB, and GAPDH mRNAs measured by PCR-based method (Hire-PAT) in HeLa cell and HCT116 cell. Gene-specific forward and reverse primers (S) amplify the region just upstream (<10bp) of poly(A) tails of target genes (top-left).

To validate the Poly(A)-seq detected poly(A) tails, we applied the Affymetrix poly(A) length assay kit based on high resolution poly(A) tail (Hire-PAT) assay [32]. We synthesized a 40-nt poly(A) tail-containing RNA using T7 polymerase [14, 23] as a control. Experimental results from the poly(A) length assay kit showed that the *in vitro* synthesized poly(A) is not uniform in length. The dominant species were around 40-nt, and a significant fraction of longer species were seen (Fig 5A). We also used the Affymetrix poly(A) length assay kit to detect the poly(A) tail lengths of mRNAs of three representative genes, including *NPM1*, *ACTB*, and *GAPDH*. It was demonstrated all the poly(A) tail lengths detected by poly(A) length assay kit were highly consistent with those detected by Poly(A)-seq (Fig 5B), thusly supporting the confidence of Poly(A)-seq-detected lengths of poly(A) tails.

### Applications of Poly(A)-seq technology on HiSeq X-ten and Nova-seq 6000

**Fig 6:**
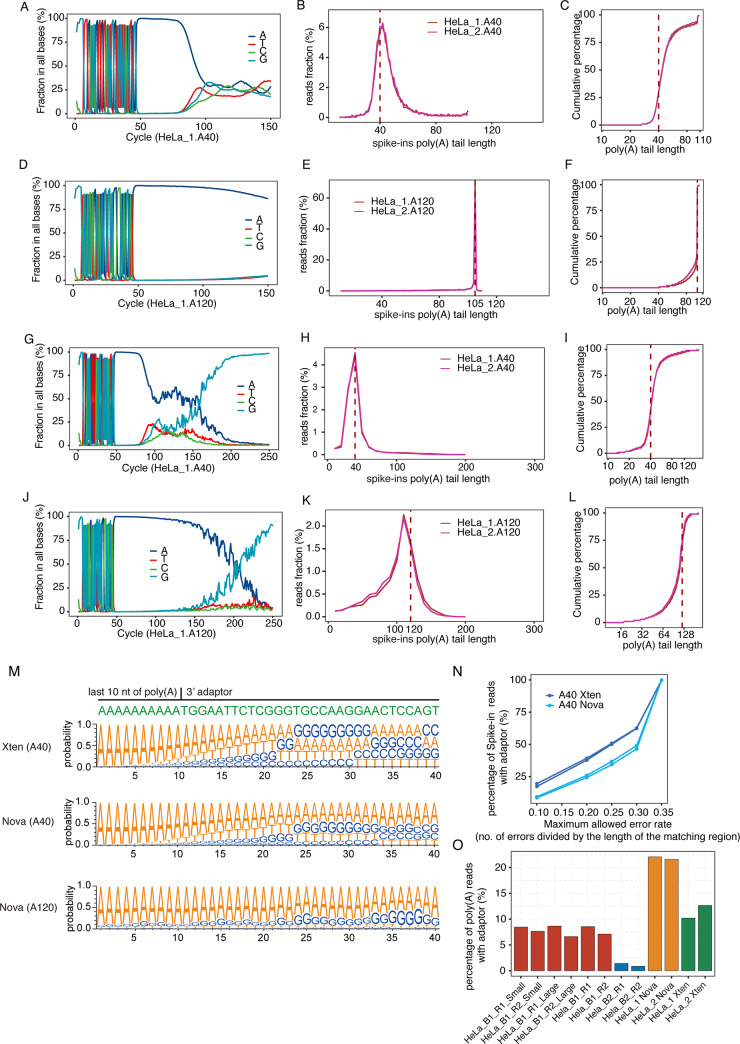
Application of Poly(A)-seq technology on HiSeq X-ten and Nova-seq 6000. A. Distribution of four different sequenced bases in each of the 150 cycle of sequencing on HiSeq X Ten for the 80-nt spike-in RNA containing 40-nt poly(A) (A40). The spike-in in HeLa_1 is shown. B. Poly(A) tail profiles of the 40-nt poly(A) spike-in. C. A cumulative fraction displaying poly(A) tail length for 40-nt poly(A) spike-in. D. Distribution of four different sequenced bases in each of the 150 cycle of sequencing on HiSeq X Ten for the 160nt spike-in RNA containing 120-nt poly(A) (A120). The spike-in in HeLa_1 is shown. Please be noted that in the 150-nt end 1 reads, the maximal detection of the poly(A) sequence is 105-nt as detailed in the main text. E. Poly(A) tail profiles of the 120-nt poly(A) spike-in. F. A cumulative fraction displaying poly(A) tail length of 120-nt poly(A) spike-in. G-L. Similar to A-F but on NovaSeq 6000, respectively. M. Sequence probability of cycles corresponding to the last 10-nt of the poly(A) tail of each spike-in and the immediately followed 30-nt adaptor sequence. N. Percentage of the spike-in reads containing the detected adaptor sequence. The results from allowing different error rates varying from 0.1, 0.2, 0.25, 0.3 and 0.35 were plotted. O. Percentage of adaptor-detectable reads among the poly(A) tail reads obtained from HeLa cells in different bench of experiments.

Due to the rapid advancement of Illumina sequencing platforms, the running cost on NextSeq 500 that we previously used to develop Poly(A)-seq has become extremely high in the recent years compared with the current popular HiSeq X-ten and Nova-seq 6000. In order to maximize the Poly(A)-seq applications, we decided to sequence Poly(A)-seq cDNA libraries on HiSeq X-ten and Nova-seq 6000. In this round of development, we prepared spike-in RNA controls by *in vitro* transcription of two RNA species, i.e. 80-nt and 160-nt RNAs containing the 40-nt and 120-nt poly(A) sequence, respectively, as well as a 40-nt of the 3’ adaptor sequence. Total RNAs extracted from HeLa cells were fragmented, followed by a purification by oligo(dT)-conjugated magnetic beads. Then, 30 pg (repeat 1) and 9 pg (repeat 2) of the purified spike-in RNA controls were added to complete library construction together (S3A Fig). Sequencing of the Poly(A)-seq libraries on HiSeq X Ten using a 150-nt pair-end mode yielded 20.3 and 19.5 millions of raw reads (end 1), among which 1.67% and 0.43% poly(A)-tail reads represented those from the 30 pg and 9 pg spike-in poly(A), respectively (S4 Table). Due to the sequence length restriction of the 150-nt pair-end mode, the maximal poly(A) tail length that were expected from the 120-nt spike-in was 105-nt, among which 104-nt of As from the poly(A) spike-in and one A from the 3’ end of the 5’ adaptor sequence. Running pA-finder resulted in a median poly(A) tail length of 65–67 nt for all genes in HeLa cells, similar to those detected by NextSeq 500 in the first bench of experiment (S3B Fig, Table 2). Meanwhile, the median poly(A) tail length for the 40-nt and 120-nt spike-in poly(A) RNAs were 44-nt and 105-nt, respectively (Fig 6B and 6E).

DNA polymerase replicates long homopolymeric sequences with decreased fidelity [31], and is thusly considered as the technical obstacle impeding the development of NGS methods of poly(A) length profiling. Additionally, a previous report of the direct reading of the poly(A) tail sequence has demonstrated the bleeding of homopolymer signal into later regions [14], which could therefore overestimate the poly(A) tail length. Our detection of a large population of the poly(A) tails longer than 40-nt could be resulted from the proposed signal bleeding (Fig 6B and 6C). No such bleeding could be detected in the 120-nt spike-in due to the restriction of the sequencing length (Fig 6E and 6F).

Taken the advantage of the known sequence of the spike-in RNAs, we were able to analyze the sequencing quality of the relative short (40-nt) and long (>100-nt) poly(A) tails, as well as the capability of the DNA polymerase in reading the adaptor sequence after the poly(A) tails. Analysis of the sequence identities of the 40-nt poly(A) tail-containing spike-in RNA revealed the first 6-nt of Gs that were added during the second strand of cDNA synthesis, which were followed by 40-nt of the 5’ adaptor sequence (unique sequence to this spike-in), 40-nt poly (A) tail, and 63-nt 3’ adaptor sequence (Fig 6A). This high consistency between the sequence profile and the spike-in sequence suggested that the DNA polymerase could successfully stop the poly(A) reading and restart the adaptor reading. To further assess this possibility, the frequency of the adaptor sequence in the 40-nt poly(A) spike-in RNA reads was analyzed. When varying the mapping stringency from allowing 10% to 35% sequencing error in the 20-nt adaptor sequence adjacent to the poly(A) tail, about 19% to 100% reads were found to contain the adaptor sequence (Fig 6N). The sequencing profile of the 120-nt poly(A) spike-in RNA showed that reading of the poly(A) signal up to 105-nt was successful (Fig 6D).

We then looked into the poly(A) sequencing accuracy and the potential effect of poly(A) signal bleeding on the adaptor reading accuracy. The quality score of poly(A) signals was constantly high for the 40-nt spike-in tail, which was sharply decreased when the DNA polymerase reaching the junction of the poly(A) tail and adaptor sequence, and kept low throughout the adaptor sequence region (S3C Fig). In the case of 120-nt spike-in tail, the poly(A) reading was at extremely high accuracy for 70-nt of As, which was gradually decreased. However, the quality score maintained as high as over 20 until 100-nt of As (S3D Fig). For the 40-nt spike-in reading, the poly(A) signal bleeding was strong at the first few adaptor positions immediately adjacent to the 3’ end of the poly(A) tails, as reflected by the high frequency of A signals. The bleeding was gradually decreased and disappeared with 10-nt of the adaptor reading, as reflected by the re-balancing of the frequency of the four different nucleotides (Fig 6M). The poly(A) signal bleeding and the low quality score in reading adaptor sequence after the 3’ end of poly(A) tails may contribute to the inaccurate identification of the poly(A) ends and also to the relative low accuracy in adaptor reading. Nevertheless, our results showed that direct sequencing of poly(A) tail on HiSeq X Ten is feasible, and the poly(A) length determination by pA-finder demonstrates an acceptable accuracy.

We next sequenced the same libraries on NovaSeq 6000 using a 250-nt pair-end mode, which allowed the read-through of the 120-nt poly(A) spike-in. As compared to HiSeq X Ten, reading of the poly(A) tails on NovaSeq 6000 showed even higher accuracy, and the drop of the quality score at the 3’ end of the poly(A) tail was even sharper for the 40-nt spike-in (S3E Fig). As for the 120-nt spike-in, the poly(A) reading until 100-nt was constantly high, which gradually dropped at the last 20-nt of As, and kept low throughout the adaptor region (S3F Fig). On the other hand, poly(A) signal bleeding was much more pronounced on the NovaSeq 600 platform than on HiSeq X Ten, and worse for the 120-nt spike-in than for the 40-nt spike-in (Fig 6G, 6J, 6M and 6N). Consistently, pA-finder using the default parameters yielded a much longer calculated poly(A) tail than the actual lengths of both spike-in species on the NovaSeq 600 platform (S4 Fig). Taken the advantage of the dramatic decrease in quality score at the junction of poly(A) tails and the 3’ adaptor, we searched for the position showing decreased sequencing quality and had it set as the 3’ end of poly(A) tail in pA-finder algorithm. The upgraded calculation effectively eliminated the effect of poly(A) signal bleeding and led to more accurate poly(A) tail lengths (Fig 6H, 6I, 6K and 6L, Table 2).

We also compared the sequencing quality profiles of Poly(A)-seq cDNA libraries obtained from HeLa cells on three different sequencing platforms, showing high sequencing quality cores for the first 120-130-nt on HiSeq X Ten and NovaSeq 6000, which was about 3-fold of that of the NextSeq 500 (S5A–S5C Fig). We also compared the frequency of the adaptor-containing reads in non-spike-in poly(A) cDNAs obtained in this study. Consistent with the observation that poly(A) signal bleeding was more severe for 120-nt than 40-nt spike-in, longer poly(A) tail-containing cDNA showed lower frequency in adaptor detecting (Fig 6O).

## Discussion

In this method paper, we present a new NGS-based method Poly(A)-seq for global profiling of poly(A) tails and measured poly(A) tail lengths, which includes the experimental method for cDNA library construction and pA-finder algorithm for poly(A) sequence identification. We have demonstrated that Poly(A)-seq was successful in direct reading of poly(A) sequence from the first cDNA strand in a 300-cycle mode on Illumina Next-seq 500. By adjusting the size of poly(A) RNA fragments and cDNA library size, we could effectively increase the frequency in detecting longer poly(A) tails. By applying Poly(A)-seq cDNA libraries containing the 40-nt and 120-nt spike-in poly(A) tails on HiSeq X-ten and NovaSeq 6000 to generate 150-nt and 250-nt pair-end reads, respectively, we demonstrate high accuracy and high quality score in reading poly(A) tails for both platforms. The poly(A) signal bleeding into the 3’ adaptor sequence and a sharp decreased quality score at the junction were observed, allowing the modification of pA-finder to calculate poly(A) tails at an adequate accuracy.

Due to the increasing appreciation of the importance of poly(A) tails in regulating translation and transcriptome [33, 34], developing NGS-based methods to profile the 3’ poly(A) tails remains a high research priority in recent years [8, 14, 21–25, 27]. Direct sequencing the poly(A) tails is attractive not only because it is straight-forward, but also for studying 3’ uridylation that controls mRNA decay and mixed tailing associated with RNA binding protein interaction and regulation [12, 33, 35, 36]. Nanopore sequencing, FLAM-seq, PAlso-seq using single molecule sequencing technologies have been applied to directly sequencing poly(A) tails from the 3ʹ-UTR into the poly(A) regions [23, 25, 27]. The Poly(A)-seq method reported here represents the first such direct sequencing approach using Illumina sequencing platforms. Given the unsurpassed sequencing depths and low sequencing price of Illumina platforms, we anticipate more broader applications of Poly(A)-seq in quantitative studies of poly(A) dynamics in the future.

Although the advantage of single molecule sequencing yielded very long reads, the detected poly(A) tails are generally shorter than 200-nt and between 50- to 100-nt for mammalian cells [25]. NovaSeq 6000 250-nt pair-end sequencing mode is a common sequencing choice among those offered by sequencing service providers, which allows the detection of over 200-nt poly(A) tail sequence (S3B Fig). We demonstrated very good quality scores during direct sequencing of poly(A) tails on both HiSeq X Ten and NovaSeq 6000. The sequence quality drops until the DNA polymerase approaching the junction between poly(A) tails and the 3’ adaptor sequence (S3C–S3F Fig). These results are in contrast to the low quality score in reading poly(A) tail from the 3’ end towards the 3ʹ-UTR region [14]. Interestingly, our finding of the sharp decrease of sequencing quality at the transition from poly(A) tails to the adaptor region is similar to the decreased fluorescence signal at the transition from poly(T) signals to the 3ʹ-UTR regions [14].

Both HiSeq X Ten and NovaSeq 6000 can resume adaptor reading immediately after poly(A) tails, however, it is noteworthy that the sequencing quality scores for the 3’ adaptor regions are drastically lower than the upstream poly(A) tails, which we could not explain at the point. The previously observed poly(A) signal bleeding [14] has also been demonstrated in this study, with more serious bleeding observed for NovaSeq 6000 reading than HiSeq X Ten, and for 120-nt poly(A) tails than the 40-nt (Fig 6M). After we added a simple parameter of identifying the position of the decreased A reading quality, the poly(A) signal bleeding was effectively removed. It is unclear yet how the poly(A) signal bleeding affects the detected poly(A) tail length by Nanopore sequencing, FLAM-seq, PAlso-seq. We noticed that the calculated spike-in poly(A) tail length was less accurate than those by TAIL-seq, likely because that we did not apply a proper model in detecting the transition site marking the sharp decreased quality score.

Although we have not analyzed in this study, Poly(A)-seq reads carry mix tailing nucleotide information as well. We have recently characterized the mix tailing of Gs in *Arabidopsis* using Poly(A)-seq reads (150-nt pair-end) obtained on HiSeq X Ten platform, which shows a functional link with the poly(A)-binding protein binding and translation efficiency [36]. Our results collectively demonstrate the feasibility of direct sequencing of poly(A) tails using Illumina sequencing platforms, which shall facilitate the study of the development and disease-related poly(A) dynamics and regulation, and of the recent emerging mixed tailing regulation.

## Supporting information

S1 Table Our data of reads & mapping statistic. (XLSX)

S2 Table Download data of reads & mapping statistic. (XLSX)

S3 Table polyA-containing reads statistic. (XLSX)

S4 Table spike-in reads statistic. (XLSX)

S1 Fig Distribution of the 5’ boundary of poly(A) regions in all poly(A)-containing reads around the annotated cleavage and polyadenylation sites (TTS). Lines with different colors indicate reads with different poly(A) tail length quantiles. Please be noted that mitochondrial reads were removed from the analysis.
(PDF)

S2 Fig Scatter plot of median poly(A) tail lengths of genes among samples in this study and among the HeLa samples obtained in this study and the previously published [14]. (TIF)

S3 Fig Poly(A)-seq on HiSeq X-ten and Nova-seq 6000. A. Electrophoresis gel images of the two cDNA libraries for Poly(A)-seq in third bench of experiments. B. Distribution of median poly(A) tail lengths in all genes expressed in HeLa cells. The Poly(A) tails in NovaSeq reads were calculated by default parameters and by adding a filter to remove the low quality As labeled as Nova QC. C. Quality scores across all bases obtained on HiSeq X Ten for the 80-nt spike-in RNA containing 40-nt poly(A) (A40). The spike-in in HeLa_1 is shown. D. Quality scores across all bases obtained on HiSeq X Ten for the 160-nt spike-in RNA containing 120-nt poly(A) (A120). The spike-in in HeLa_1 is shown. E-F. Same as in C-D, respectively, except for the scores were those obtained on NovaSeq 6000.
(PDF)

S4 Fig Poly(A) tail profiles of poly(A) spike-in calculated by pA-finder using default parameters on the NovaSeq 600 platform. A. Poly(A) tail profiles of the 40-nt poly(A) spike-in. B. A cumulative fraction displaying poly(A) tail length for 40-nt poly(A) spike-in. C. Poly(A) tail profiles of the 120-nt poly(A) spike-in. D. A cumulative fraction displaying poly(A) tail length for 40-nt poly(A) spike-in.
(PDF)

S5 Fig Sequencing quality profiles for reading Poly(A)-seq cDNA libraries on three different sequencing platforms. A. Quality scores across all bases obtained on NextSeq 500 for HeLa_B1_R1. B. Quality scores across all bases obtained on HiSeq X Ten for HeLa_1. C. Quality scores across all bases obtained on NovaSeq 6000 for HeLa_1.
(PDF)
