# Supplementary figures and images for: Poly(A)-seq: A method for direct sequencing and analysis of the transcriptomic poly(A)-tails
Source: PLoS One. 2020 Jun 16;15(6):e0234696. doi: 10.1371/journal.pone.0234696 (PMC7297374; doi:10.1371/journal.pone.0234696)

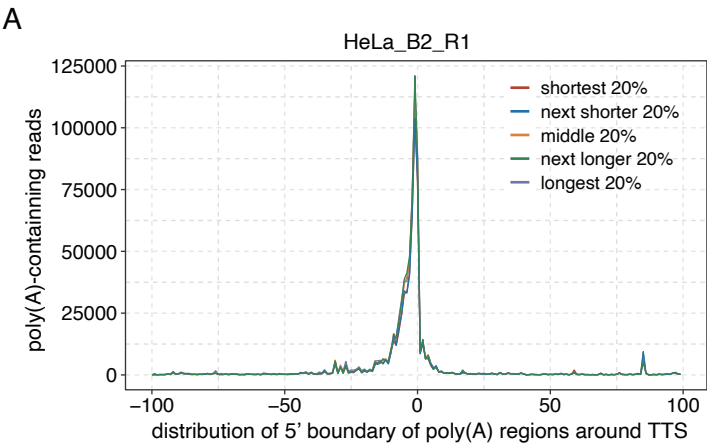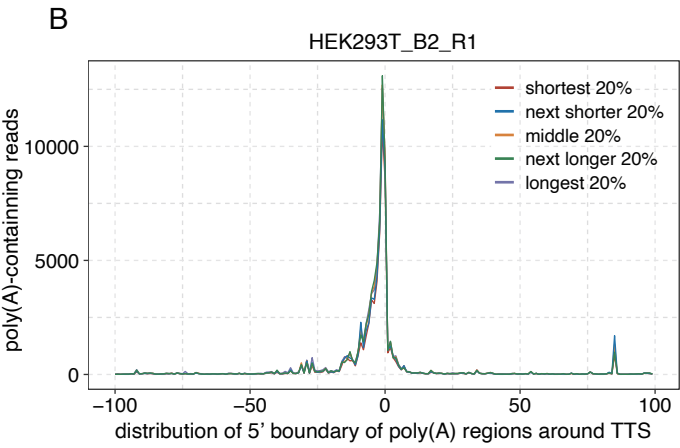

Supplement: S1 Fig — Lines with different colors indicate reads with different poly(A) tail length quantiles. Please be noted that mitochondrial reads were removed from the analysis. (PDF) [file pone.0234696.s005.pdf]

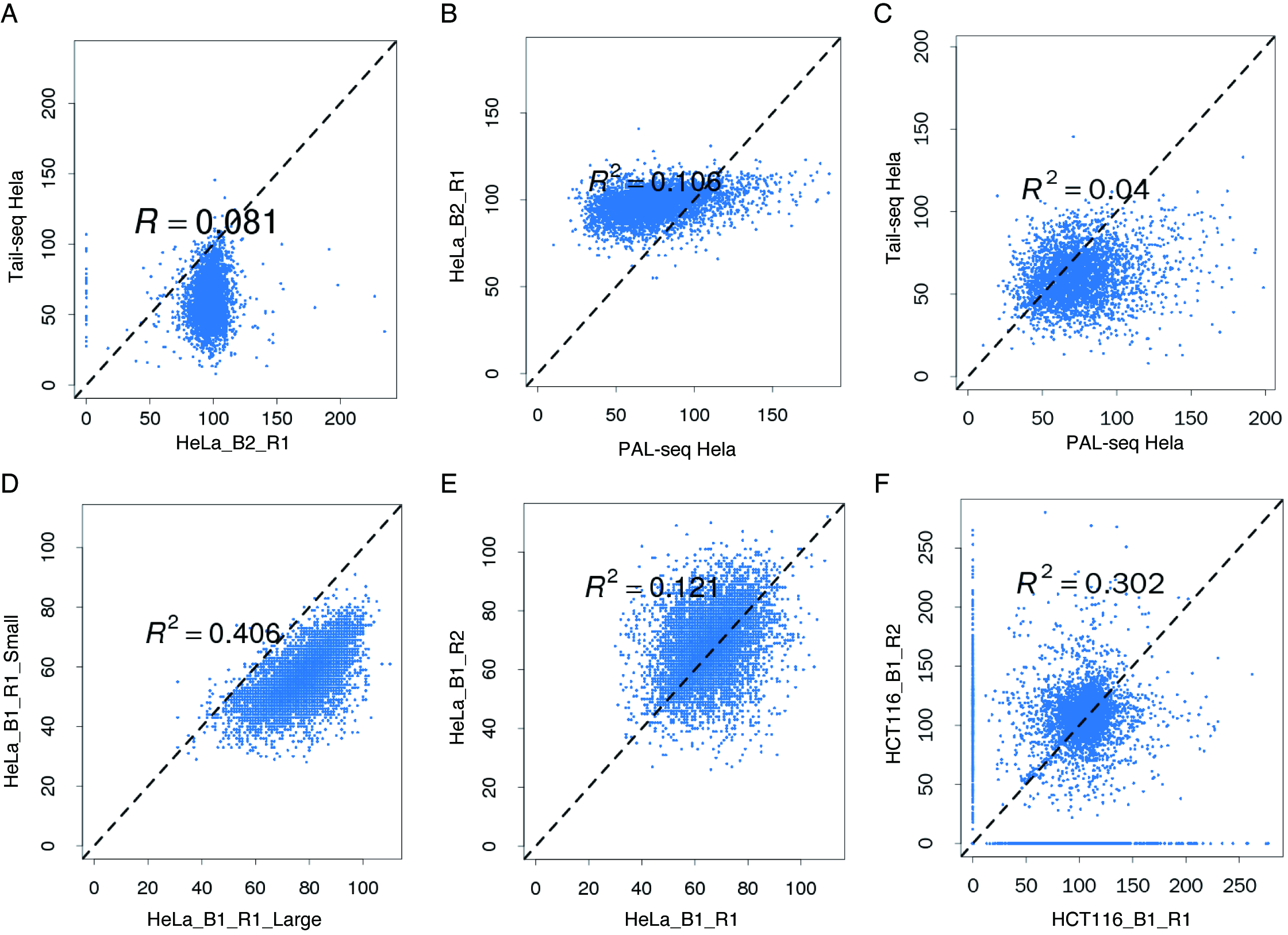

Supplement: S2 Fig — (TIF) [file pone.0234696.s006.tif]

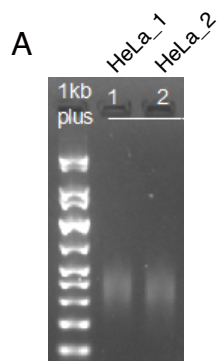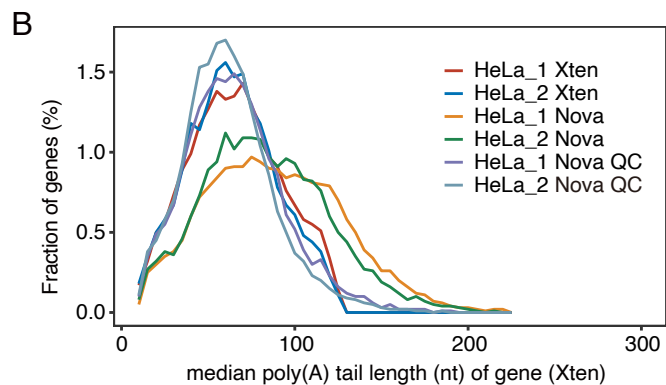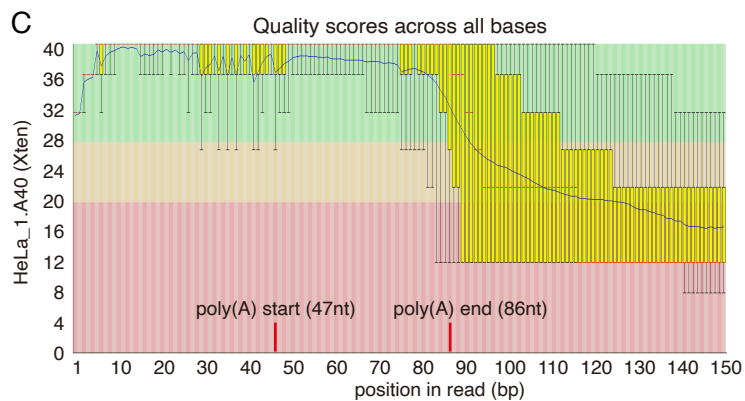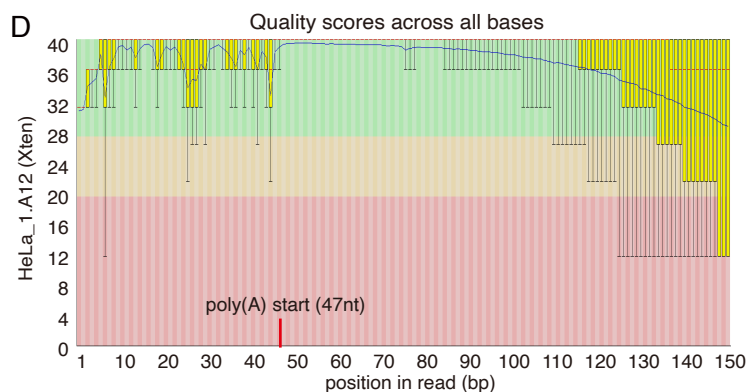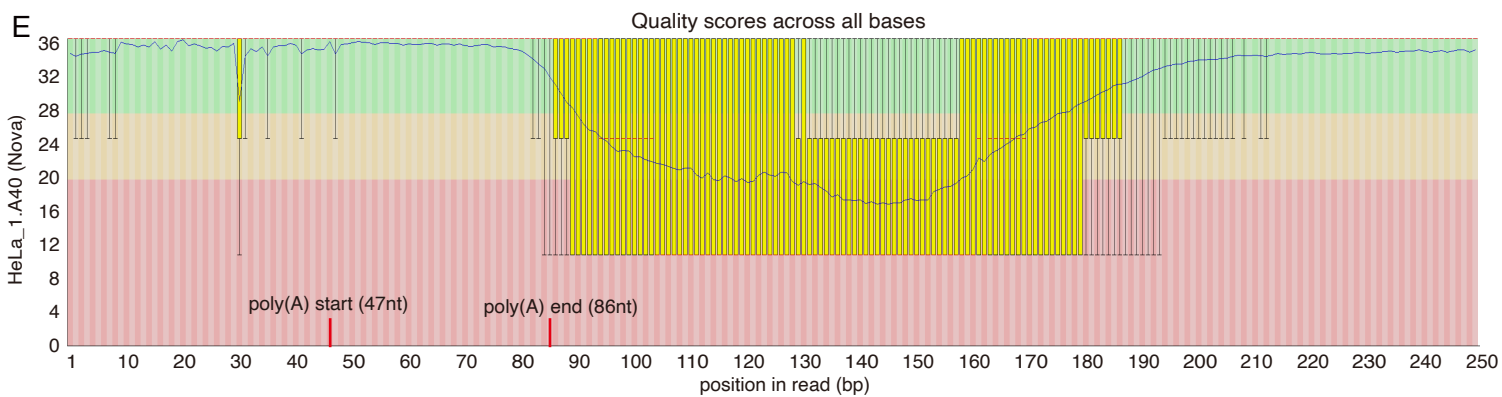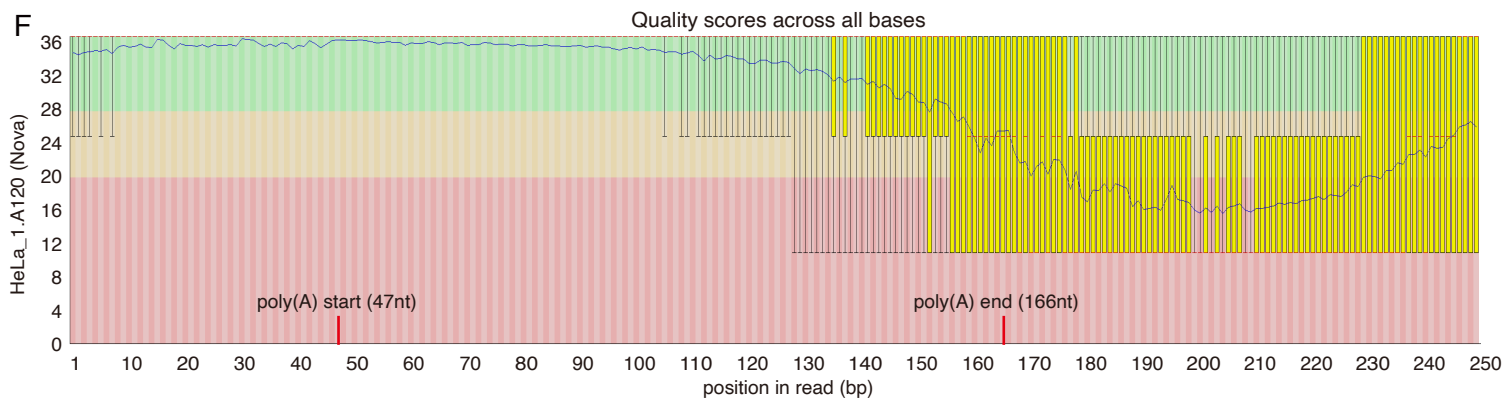

Supplement: S3 Fig — A. Electrophoresis gel images of the two cDNA libraries for Poly(A)-seq in third bench of experiments. B. Distribution of median poly(A) tail lengths in all genes expressed in HeLa cells. The Poly(A) tails in NovaSeq reads were calculated by default parameters and by adding a filter to remove the low quality As labeled as Nova QC. C. Quality scores across all bases obtained on HiSeq X Ten for the 80-nt spike-in RNA containing 40-nt poly(A) (A40). The spike-in in HeLa_1 is shown. D. Quality scores across all bases obtained on HiSeq X Ten for the 160-nt spike-in RNA containing 120-nt poly(A) (A120). The spike-in in HeLa_1 is shown. E-F. Same as in C-D, respectively, except for the scores were those obtained on NovaSeq 6000. (PDF) [file pone.0234696.s007.pdf]

A

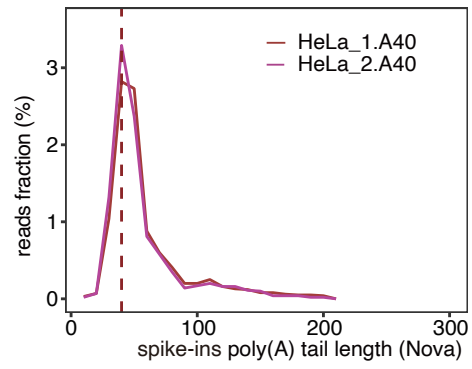

B

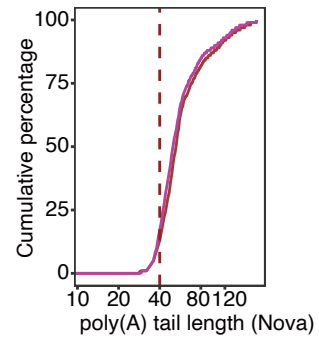

C

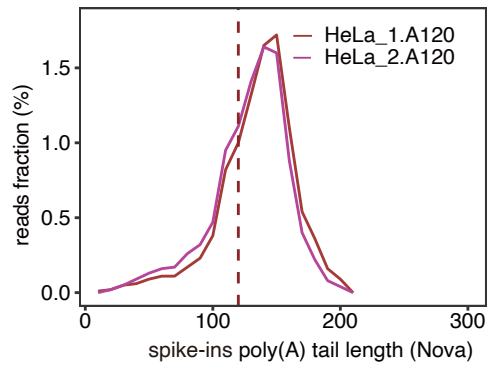

D

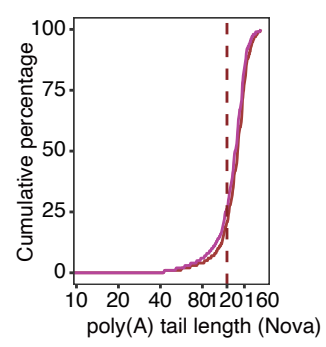

Supplement: S4 Fig — A. Poly(A) tail profiles of the 40-nt poly(A) spike-in. B. A cumulative fraction displaying poly(A) tail length for 40-nt poly(A) spike-in. C. Poly(A) tail profiles of the 120-nt poly(A) spike-in. D. A cumulative fraction displaying poly(A) tail length for 40-nt poly(A) spike-in. (PDF) [file pone.0234696.s008.pdf]

A

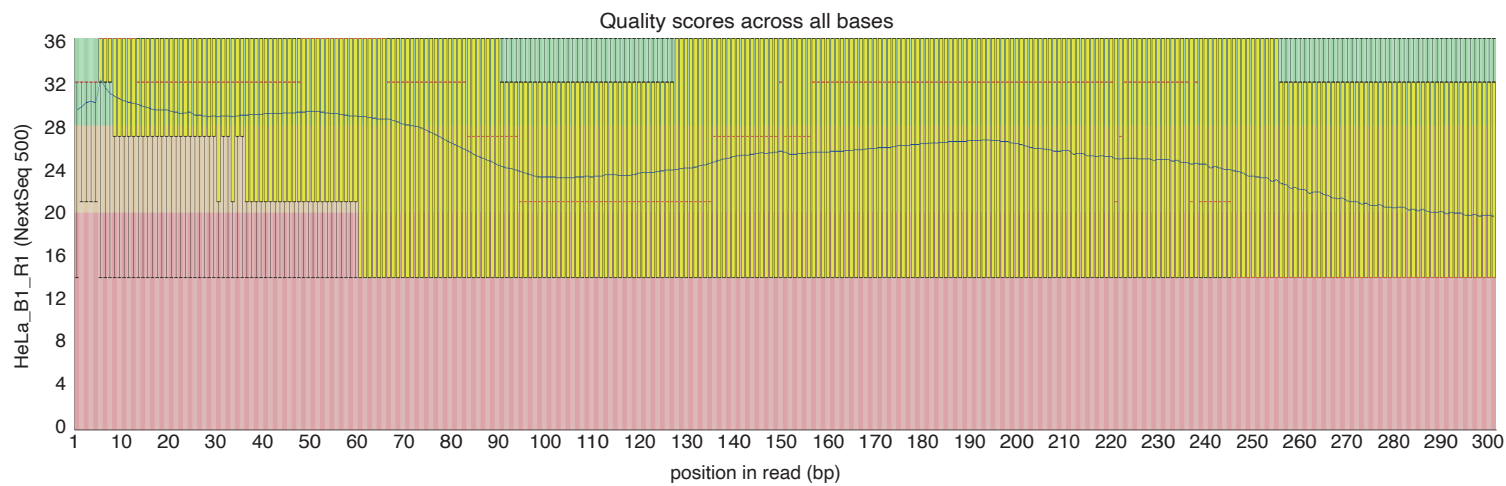

B

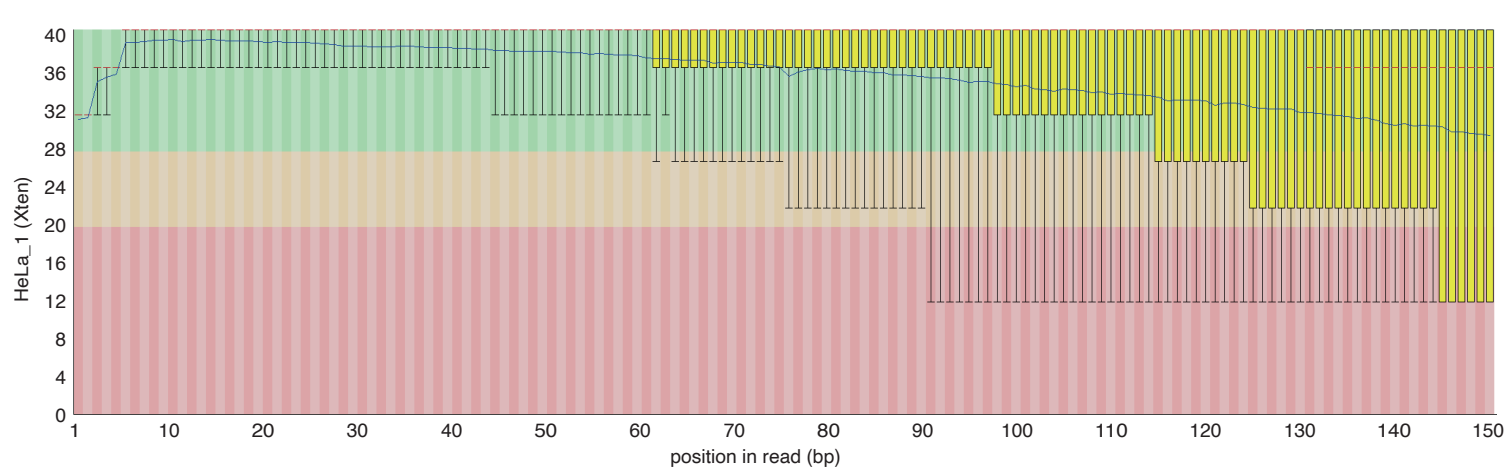

C

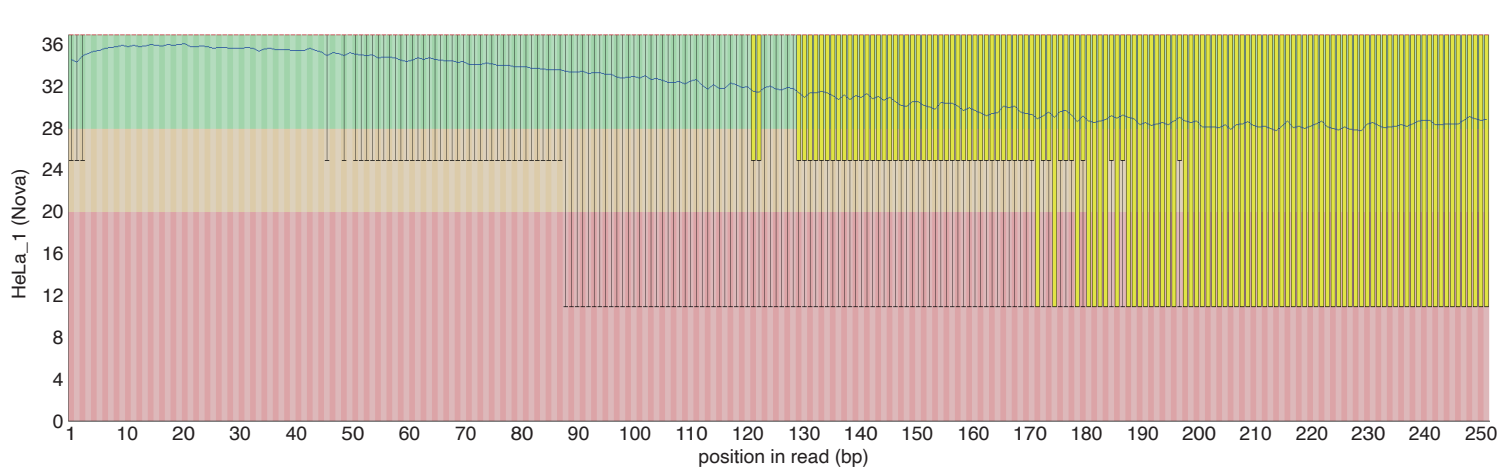

Supplement: S5 Fig — A. Quality scores across all bases obtained on NextSeq 500 for HeLa_B1_R1. B. Quality scores across all bases obtained on HiSeq X Ten for HeLa_1. C. Quality scores across all bases obtained on NovaSeq 6000 for HeLa_1. (PDF) [file pone.0234696.s009.pdf]
